# A Novel Look at Dosage-Sensitive Sex Locus Xp21.2 in a Case of 46,XY Partial Gonadal Dysgenesis without *NR0B1* Duplication

**DOI:** 10.3390/ijms24010494

**Published:** 2022-12-28

**Authors:** Ana Paula Francese-Santos, Jakob A. Meinel, Cristiane S. C. Piveta, Juliana G. R. Andrade, Beatriz A. Barros, Helena Fabbri-Scallet, Vera Lúcia Gil-da-Silva-Lopes, Gil Guerra-Junior, Axel Künstner, Hauke Busch, Olaf Hiort, Maricilda P. de Mello, Ralf Werner, Andréa T. Maciel-Guerra

**Affiliations:** 1 Department of Translational Medicine, State University of Campinas (UNICAMP), Campinas 13083-888, SP, Brazil; 2 Molecular Biology and Genetic Engineering Center, State University of Campinas (UNICAMP), Campinas 13083-875, SP, Brazil; 3 Department of Pediatrics and Adolescent Medicine, Division of Paediatric Endocrinology and Diabetes, University of Lübeck, 23562 Lübeck, Germany; 4 Interdisciplinary Group for the Study of Sex Determination and Differentiation (GIEDDS), State University of Campinas (UNICAMP), Campinas 13083-888, SP, Brazil; 5 Department of Pediatrics, State University of Campinas (UNICAMP), Campinas 13083-888, SP, Brazil; 6 Group of Medical Systems Biology, Lübeck Institute of Experimental Dermatology, University of Lübeck, 23562 Lübeck, Germany; 7 Institute of Molecular Medicine, University of Lübeck, 23562 Lübeck, Germany

**Keywords:** disorders of sex development, 46,XY partial gonadal dysgenesis, Xp21.2 duplication, *NR0B1*, chromosome microarray analysis, whole-genome sequencing

## Abstract

A region of 160 kb at Xp21.2 has been defined as dosage-sensitive sex reversal (DSS) and includes the *NR0B1* gene, considered to be the candidate gene involved in XY gonadal dysgenesis if overexpressed. We describe a girl with 46,XY partial gonadal dysgenesis carrying a 297 kb duplication at Xp21.2 upstream of *NR0B1* initially detected by chromosomal microarray analysis. Fine mapping of the breakpoints by whole-genome sequencing showed a tandem duplication of *TASL (CXorf21)*, *GK* and partially *TAB3*, upstream of *NR0B1*. This is the first description of an Xp21.2 duplication upstream of *NR0B1* associated with 46,XY partial gonadal dysgenesis.

## 1. Introduction

46,XY partial gonadal dysgenesis (GD) is a non-syndromic form of incomplete testicular development, belonging to the Disorders/Differences of Sex Development (DSD) condition. Clinically, partial GD is characterized by genital ambiguity due to variable degrees of testicular failure in individuals with a 46,XY karyotype. Pathogenic variants have been identified in many genes involved in sex determination, however, in most cases the etiology remains unknown [1,2].

Duplications in the short arm of the X chromosome involving *NR0B1* (Nuclear Receptor Subfamily 0, Group B, Member 1) have been associated with 46,XY DSD. Initially detected by karyotyping [3], these duplications were further characterized by fluorescent in situ hybridization (FISH) [4] and chromosome microarray analysis (CMA) and/or multiplex ligation-dependent probe amplification (MLPA) [5,6]. A 160 kb region of Xp21.2 has been defined as the critical region responsible for sex reversal [3]; this area is known as dosage-sensitive sex reversal (DSS), and it is believed that the *NR0B1* gene contained in that region causes sex reversal in a dosage-dependent manner [5,6].

Though current data support the hypothesis that *NR0B1* is responsible for sex reversal if overexpressed, *NR0B1* single duplication associated with 46,XY GD is still to be demonstrated as evidence of its direct involvement in this condition, and the role of other genes and regulatory regions mapped to the Xp21 may not be completely ruled out [6].

Here, we describe a girl with 46,XY partial GD with a 297 Kb duplication at Xp21.2 upstream of *NR0B1* identified by CMA. The duplication was further characterized by whole-genome sequencing (WGS).

## 2. Detailed Case Description

### 2.1. Patient

Our patient was the second child of healthy unrelated parents and was referred to the DSD service at 5 days old due to genital ambiguity. Delivery was full-term after an uneventful pregnancy with birth weight of 3410 g and length of 49 cm. The older brother was a healthy child with typical male genitalia. Physical examination revealed a 0.5 cm phallus, a single perineal opening, partially fused labioscrotal folds and nonpalpable gonads (External Masculinization Score—EMS = 4) [7]. There was no dysmorphic picture associated with genital ambiguity.

Karyotype was 46,XY. At 1 month old, hormonal evaluation revealed high levels of follicle-stimulating hormone (FSH) (24 IU/L; normal range (NR): 1.5 to 12.4 IU/L) and luteinizing hormone (LH) (10 IU/L; NR: 1.7 to 8.6 IU/L) and low testosterone (0.2 ng/mL; NR: 2.86 to 8.10 ng/mL) with normal levels of testosterone precursors (progesterone, 17-OH-progesterone, androstenedione and dehydroepiandrosterone). No abnormalities were identified on abdominal ultrasound; in turn, genitography showed a urogenital sinus.

The infant was assigned female. When the girl was 1 year old, she underwent bilateral gonadectomy and introitoplasty. Laparoscopy revealed absence of the uterus and cystoscopy showed a blindly ending vagina; left gonadal tissue was absent and there was a right dysgenetic testis with some areas of fibrous tissue surrounding immature seminiferous tubules without spermatogonia. The clinical, hormonal and histological picture led to the diagnosis of 46,XY partial GD.

On follow-up, she had normal neuromotor development, no learning disabilities, no significant health problems and normal growth velocity; her height was between the 90th and 95th centile for a girl. At 11.5 years estrogen replacement began, and imaging studies confirmed the absence of the uterus. Two years later, breast development was complete and final height was 174 cm.

She returned to our DSD service when she was 20 years old because another introitoplasty was required, followed by the use of vaginal dilators. At that time, genetic investigation and clinical follow-up were resumed, as well as. Evaluation of adrenal function showed normal results, and at the last visit to our service at the age of 26 years there were normal levels of adrenocorticotropic hormone (ACTH) (19.6 pg/mL; NR: 7.20 to 63.3 pg/mL) and cortisol (12.1 µg/dL; NR: 6.20 to 19.40 µg/dL).

### 2.2. Genetic Studies

Chromosomal microarray analysis (CMA) (GeneChip CytoScan^®^ 750, Affymetrix Inc.) was performed, followed by whole-genome sequencing (WGS) (Supplementary Material).

### 2.3. Results

**Figure 1:**
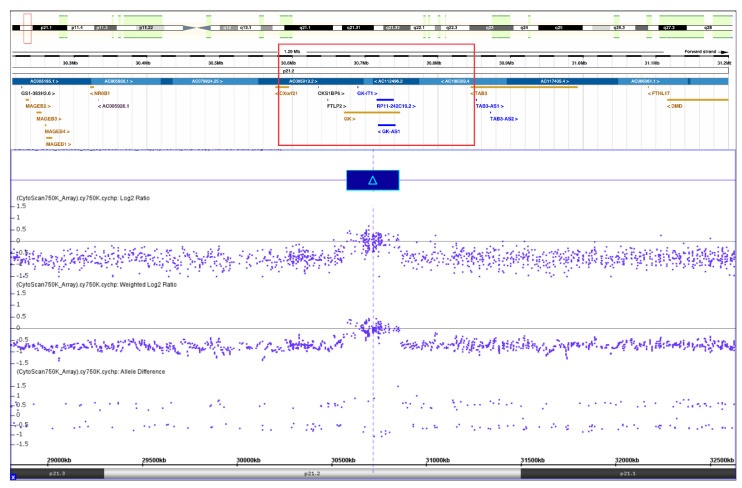
Chromosome microarray analysis showing duplication of approximately 277kb at Xp21.2 not covering the *NR0B1* gene.

**Figure 2:**
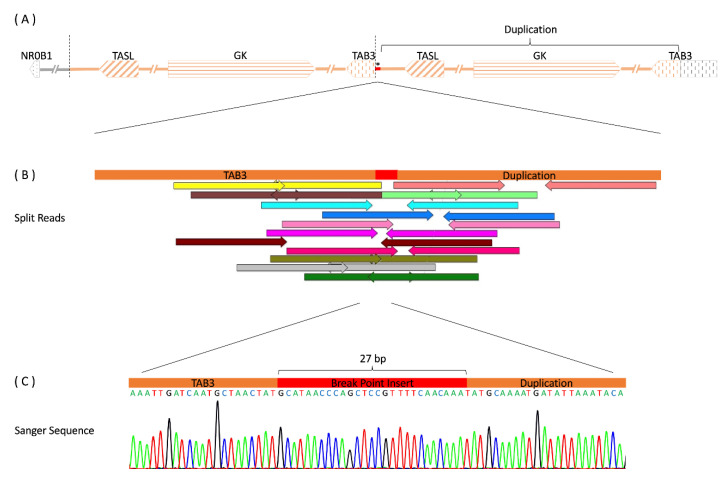
Structural variation of our patient (**A**) Overview of the copy number variation (CNV) upstream of the *NR0B1* gene and its orientation within the Xp21.2 region. Arrows depict genes and their respective direction of transcription. The chromosome segment is drawn with the distal chromosome arm to the left and the centromere to the right. The distances between genes are not to scale. The region chrX: 30,561,644-30,859,140 has been duplicated in a tandem manner, as indicated by the segments shaded orange. * marks a 27 bp insert at the breakpoint. (**B**) Position of extracted and aligned split reads from genome sequencing crossing the breakpoint (chrX:30,859,140). Correspondingly colored arrows of opposite directions belong to the same read pair. Read pairs have been mapped 297.5 kb away from each other. (**C**) Verification of the breakpoint sequence by PCR and Sanger sequencing.

CMA revealed a ≈ 277 kb duplication at Xp21.2 (30,30–187,187–580,580–693,857) (Figure 1) which was inherited from the mother. WGS confirmed this structural variation showing a 297 kb duplication including *GK* (glycerol kinase) and *TASL* (TLR adaptor interacting with endolysosomal SLC15A4, previously known as CXorf21), together with their respective promoter and predicted enhancer regions, as well as the 3’-end of *TAB3* (TGF-beta-activated kinase 1 binding protein 3). Duplication borders were apparent through increased read-depth and split reads mapping 297 kb apart. Paired split reads were extracted and aligned to generate a continuous sequence of the breakpoints showing a tandem duplication, where intron 9 of *TAB3* is merged to the downstream region of *TASL* by a 27 bp insert. The sequences at the breakpoints were confirmed by polymerase chain reaction (PCR) and Sanger sequencing (Figure 2).

Only two heterozygous missense single nucleotide variants (SNVs) were found in protein-coding regions of genes related to 46,XY DSD: the c.361C > T, p.(Arg121Trp) variant in *STAR* (steroidogenic acute regulatory protein) and the c.1891G > A, p.(Val631Ile) variant in *POR* (cytochrome p450 oxidoreductase), neither pathogenic nor likely pathogenic. Both genes lead to autosomal recessive steroid biosynthesis deficiencies and were excluded as a putative cause of this patient’s clinical picture.

## 3. Discussion

In the present case, partial testicular differentiation did not affect early anti-Müllerian hormone (AMH) secretion by Sertoli cells, as shown by the absence of uterus and upper vagina; however, it led to severe impairment of the Leydig cells’ function, as indicated by the low degree of masculinization of the external genitals. Complete regression of the testicular tissue occurred further in the left gonad, and a small amount of immature testicular tissue remained on the right side.

The Xp21.2 duplication is the only likely cause of 46,XY partial GD we could identify in our patient since WGS excluded any other major known causes of DSD. The 297 Kb tandem duplication included an extra dose of *GK* and *TASL* and partial duplication of *TAB3*. However, it did not include *NR0B1*, which is the strongest candidate to cause GD in Xp21.2 duplications.

Regarding the three genes included in this duplication, the *GK* is mainly expressed in the liver, skeletal muscle, kidney and brain. The protein encoded by this gene, glycerol kinase, is a critical enzyme in regulating glycerol uptake and metabolism [8]. In turn, *TASL* is involved in initiating immune responses and is mainly expressed in the bone marrow and lymphoid tissue [9]. *TAB3* is a constituent of the NF-kappaB pathway, a protein complex that controls the transcription of DNA, cytokine production and cell survival; the product of this gene is expressed in the testis, but has low tissue specificity [10]. There has been no association of these genes with DSD other than their presence inside the DSS region.

The absence of *NR0B1* in the duplicated segment caught our attention. Normal testicular development requires a precise amount of its expression. This gene was initially designated as *DAX1* (dosage-sensitive sex reversal, adrenal hypoplasia critical region, on chromosome X, gene 1) and encodes an orphan member of the nuclear receptor superfamily, a protein still referred to as DAX1 [11]. During sex determination, DAX1 seems to acts as a dominant-negative regulator that interacts with genes involved in the development of the hypothalamic-pituitary-adrenal-gonadal axis and in the biosynthesis of steroid hormones; however, its exact biological role remains unclear [12,13].

Mutations or deletions of *NR0B1* cause X-linked congenital adrenal hypoplasia, characterized by primary adrenal insufficiency, hypogonadotropic hypogonadism and impaired fertility; however, boys have normal testicular development at birth [14], making *NR0B1* dispensable for male external development. In turn, Xp duplications involving *NR0B1* have been associated with both partial and complete 46,XY GD, the latter being characterized by streak gonads and normal internal and external female genitalia [5,6,15,16]. In normal XY individuals, *NR5A1* interacts with *SRY* and later *SOX9* to upregulate *Sox9*-driving SF1^+^-supporting cell precursors into the Sertoli cell line [17], allowing development of the male gonad. When Xp duplication occurs, overexpression of *NR0B1* disrupts *NR5A1* binding to its DNA targets and prevents coactivation at the *Sox9* promoter and enhancers [18,19] leading to dysgenetic testes, streak gonads or sex reversal in mice [4,5,16,20].

A patient with complete XY GD with two duplications and two small deletions that did not include *NR0B1* was recently described [21]. High-throughput chromosome conformation capture (Hi-C) analysis of this case revealed rearrangement of a topological-associated domain (TAD) boundary close to *NR0B1* which was associated with neo-TAD formation that may cause enhancer hijacking and ectopic *NR0B1* expression. Interestingly, the proposed ectopic enhancers of *TASL* and *GK* are also duplicated in this case of partial GD, strengthening the role of these regulatory regions. To our knowledge, no similar case has been described so far in patients with 46,XY partial GD.

## 4. Conclusions

In summary, though *NR0B1* is the most likely candidate gene for 46,XY GD in the DSS region, the *Xp21*.2 duplication which does not include *NR0B1* is the only identifiable cause in this case. Thus, we hypothesize that genomic imbalance brought on by this duplication has led to the disruption of specific regulatory elements and dysregulation of *NR0B1* expression.

## Supplementary Materials

The following supporting information can be downloaded at: https://www.mdpi.com/article/10.3390/ijms24010494/s1.
