# Supplementary material for: A Novel Look at Dosage-Sensitive Sex Locus Xp21.2 in a Case of 46,XY Partial Gonadal Dysgenesis without NR0B1 Duplication
Source: Int J Mol Sci. 2022 Dec 28;24(1):494. doi: 10.3390/ijms24010494 (PMC9820669; doi:10.3390/ijms24010494)
Supplement: Supplementary file 1 [file ijms-24-00494-s001.zip › ijms-1982951-supplementary.pdf]

Supplementary Material

Chromosomal microarray analysis

DNA samples were hybridised onto the *GeneChip CytoScan®* 750 *Array Kit* (Affymetrix Inc., Santa Clara, California, USA) and processed as recommended by the manufacturer. Array data were analysed using Chromosome Analysis Suite (ChAS) (Affymetrix ®). Standard analysis adopted the following parameters: markers >25 for deletion; >50 for duplication, without filter size for CNVs. Data were analysed using CytoGenomics Software version 4.0.2.21 (Agilent) and the Database of Genomic Variants (DGV, Version CNV_DGV_hg19_v4, Toronto, Canada).

Whole genome sequencing

WGS has been performed to fine-map the breakpoints of the duplications in Xp21.2. Sequencing libraries were constructed from 1.0 µg DNA per sample using the Truseq Nano DNA HT Sample preparation Kit (Illumina, USA) following the recommendations of the manufacturer. Genomic DNA was randomly fragmented to a size of approximately 350 bp by Covaris cracker (Covaris, USA). DNA fragments were blunted, A-tailed and ligated with the full-length adapter for Illumina sequencing with further PCR amplification. Libraries were purified using AMPure XP (Beckman Coulter, USA), analysed for size distribution on an Agilent 2100 Bioanalyser (Agilent Technologies) and quantified by Q-PCR. Paired-end sequencing of libraries was performed on Illumina HiSeq platforms (Illumina). Per sample more than 90 GB of raw data were obtained, resulting in an average read depth of 30x.

Sequencing reads were mapped to human reference genome version GRCh37/hg19 using Burrows-Wheeler Aligner (Li and Durbin 2009). The resulting mapping files were screened for duplicated reads applying *Picard* tools *MarkDuplicates* version 1.111

(Picard: http://sourceforge.net/projects/picard/). Split-reads and discordant paired-end alignments were extracted using SAMtools version 0.1.18 (Li and Durbin 2009). SNPs and InDels were called as implemented in Genome Analysis Toolkit (GATK) version 3.8.0 (DePristo et al. 2011) SV detection and CNV detection were performed using DELLY and control-FREEC (Boeva et al. 2012; Rausch et al. 2012). Variations were annotated using ANNOVAR (Wang, Li, and Hakonarson 2010). All chromosomal positions in this paper are according to GRCh37.

Breakpoint Sequencing

Primers at either end of the constructed breakpoint sequences were designed using Lasergene PrimerSelect (DNASTAR, Wisconsin, USA). Optimal annealing temperature was determined through gradient PCR and electrophoresis. Consequently, PCR setup was 35 cycles with 30 sec. at 95°C for denaturation, 30 sec. at primer specific temperature for annealing and 1 min. extension at 72°C. Sanger sequencing of amplicons was performed on a 3130 Genetic Analyzer (Applied Biosystems, Foster City, USA).
